# Oxidative stress biomarkers and chondrocyte apoptosis as predictors of postoperative outcomes in osteonecrosis of the femoral head

**DOI:** 10.5937/jomb0-61682

**Published:** 2026-03-17

**Authors:** Chia-Jui Hu, Ming Song, Lai Yu, Guanfei Zeng

**Affiliations:** 1 Xiamen Chang Gung Hospital, Department of Orthopaedics, Xiamen, China

**Keywords:** osteonecrosis of the femoral head, oxidative stress, biomarkers, chondrocyte apoptosis, prognosis, medical biochemistry, osteonekroza glave femura, oksidativni stres, biomarkeri, apoptoza hondrocita, prognoza, medicinska biohemija

## Abstract

**Background:**

Osteonecrosis of the femoral head (ONFH) is a progressive disease where oxidative stress and chondrocyte apoptosis contribute to cartilage degeneration and postoperative complications. Identifying reliable biochemical markers is essential for prognosis after hip resurfacing arthroplasty (HRA).

**Methods:**

A retrospective analysis was conducted in 134 ONFH patients (ARCO stage II-III) treated with HRA. Serum oxidative stress markers-reactive oxygen species (ROS), superoxide dismutase (SOD), and malondialdehyde (MDA)-were measured preoperatively and at serial postoperative timepoints. Chondrocyte apoptosis rates were assessed by flow cytometry. Pain and functional recovery were evaluated using VAS and Harris hip scores. Correlation, univariate, and Cox regression analyses were applied to determine associations with postoperative outcomes.

**Results:**

Patients with high oxidative stress had significantly increased ROS and MDA levels, reduced SOD activity, and elevated chondrocyte apoptosis (P&lt;0.05). These patients showed delayed functional recovery and higher complication rates. Strong correlations were observed between oxidative markers and apoptosis (ROS: r=0.912; MDA: r=0.901; SOD: r=-0.875). Cox regression identified ROS, MDA, reduced SOD, and apoptosis as independent risk factors for postoperative failure (HR range: 1.06-1.49, all P&lt;0.05).

**Conclusions:**

Serum oxidative stress markers and chondrocyte apoptosis rates are closely linked to joint recovery and complications after HRA in ONFH patients. These biochemical indicators may serve as prognostic biomarkers, supporting early identification of high-risk individuals and guiding personalized postoperative interventions.

## Abstract (sr)

**Uvod:**

Osteonekroza glave femura (ONFH) je progresivna bolest gde oksidativni stres i apoptoza hondrocita doprinose degeneraciji hrskavice i postoperativnim komplikacijama. Identifikacija pouzdanih biohemijskih markera je neophodna za prognozu nakon artroplastike kuka obnovom površine (HRA).

**Metode:**

Retrospektivna analiza je sprovedena kod 134 pacijenta sa ONFH (ARCO stadijum II-III) lečenih HRA. Serumski markeri oksidativnog stresa - reaktivne vrste kiseonika (ROS), superoksid dismutaza (SOD) i malondialdehid (MDA) - mereni su preoperativno i u serijskim postoperativnim vremenskim tačkama. Stope apoptoze hondrocita procenjene su protočnom citometrijom. Bol i funkcionalni oporavak procenjeni su korišćenjem VAS i Haris skale za kukove. Korelaciona, univarijantna i Koksova regresiona analiza primenjene su da bi se utvrdile povezanosti sa postoperativnim ishodima.

**Rezultati:**

Pacijenti sa visokim oksidativnim stresom imali su značajno povećane nivoe ROS i MDA, smanjenu aktivnost SOD i povišenu apoptozu hondrocita (P&lt;0,05). Ovi pacijenti su pokazali odloženi funkcionalni oporavak i veće stope komplikacija. Primećene su jake korelacije između oksidativnih markera i apoptoze (ROS: r=0,912; MDA: r=0,901; SOD: r=-0,875). Koksova regresija je identifikovala ROS, MDA, smanjeni SOD i apoptozu kao nezavisne faktore rizika za postoperativni neuspeh (HR opseg: 1,06-1,49, sve P&lt;0,05).

**Zaključak:**

Serumski markeri oksidativnog stresa i stope apoptoze hondrocita su usko povezani sa oporavkom zglobova i komplikacijama nakon HRA kod pacijenata sa ONFH. Ovi biohemijski indikatori mogu poslužiti kao prognostički biomarkeri podržavajući ranu identifikaciju osoba sa visokim rizikom i usmeravajući personalizovane post - operativne intervencije.

## Introduction

Osteonecrosis of the femoral head (ONFH) is an ischemic necrosis of bone tissue caused by the interruption or reduction of blood supply to the femoral head. Its pathological mechanism is complex and involves multiple factors, including vascular impairment, inflammatory response, oxidative stress, and apoptosis [1][2]. Hip resurfacing arthroplasty (HRA) has been widely applied as a bone-preserving surgical option for functional reconstruction in early and middle stages of ONFH, particularly in younger patients [3]. Despite its clinical advantages, the incidence of postoperative failure and long-term complications remains high, and the molecular mechanisms underlying these outcomes have not been fully elucidated.

Oxidative stress has emerged as a central contributor to the pathological microenvironment of joints [4]. Elevated reactive oxygen species (ROS) can impair cellular homeostasis, promote chondrocyte apoptosis, and accelerate cartilage destruction, thereby affecting cartilage repair after HRA and exacerbating degeneration of periprosthetic tissue [5]. In addition, failure risk is influenced by prosthesis loosening, infection, and insufficient recovery of cartilage function [6]. Since the interplay of oxidative stress and chondrocyte apoptosis involves surgical stress, material-related factors, and inflammatory regulation, clarifying their roles in HRA failure is clinically relevant [7].

Currently, there is a lack of targeted laboratory indicators to predict postoperative outcomes. Oxidative stress-related markers and apoptosis indices may serve as promising prognostic biomarkers [8]. For example, changes in antioxidant enzymes such as superoxide dismutase (SOD) and glutathione peroxidase (GPx) have been linked to postoperative inflammation and cartilage degeneration [9], while alterations in apoptosis-related proteins including Caspase-3 and Bcl-2 may reflect periprosthetic cartilage function [10].

Based on this rationale, the present study aimed to systematically evaluate the association between oxidative stress markers and chondrocyte apoptosis in ONFH patients after HRA, and to determine their impact on postoperative outcomes. Emphasizing biochemical and cellular indicators, this work seeks to provide a theoretical basis for developing prognostic biomarkers and personalized postoperative management strategies.

## Materials and methods

### General data

From January 2021 to June 2023, this study retrospectively analyzed 134 patients with ONFH who had been treated in three hospitals in Eastern United States region, and the patients had been de-identified and only the existing clinical data were analyzed. (1) Inclusion criteria: It meets the diagnostic criteria of stage II-III of the association research circulation osseous (ARCO); Aged between 30-60 years with a body mass index (BMI) of 18.5-28 kg/m^2^; No serious dysfunction of heart, lung, kidney and other important organs; No medication affecting the level of oxidative stress (such as antioxidants and anti-inflammatory drugs) was taken before surgery; Preoperative imaging showed no severe cartilage destruction or femoral head collapse >5 mm. (2) Exclusion criteria: There were 17 cases combined with other types of hip joint diseases (such as hip joint dysplasia or hip joint inflammation); There were 12 cases with obvious infection or inflammatory reaction (C-reactive protein >10 mg/L, and white blood cell count >10x10^9^/L) before surgery; Combined with hematological diseases or malignant tumors (8 cases); Pregnant women or nursing women (6 cases); 5 cases were withdrawn from the study due to other reasons during the follow-up period.

### Methods

### Therapeutic method

The patient underwent HRA. Preoperative frontal and lateral radiographs and MRI of the hip were routinely performed to assess the extent of ONFH and cartilage status. The surgery was performed under general anesthesia by the same professional team, with a prosthesis from the Birmingham Hip Resurfacing system (BHR system) manufactured by Smith & Nephew, USA, being used. During the surgery, the original bone mass as much as possible was preserved through debridement of the femoral head, and the joint function was reconstructed with metal surface covering. The prosthesis was fixed with bone cement (Palacos R+G bone cement from Zimmer Biomet) with a cement layer thickness of 2-3 mm. The position and fixation of the prosthesis were confirmed postoperatively using a C-arm X-ray machine (Philips BV Model: Pulsera). Intraoperative infection prevention measures included intravenous ceftriaxone sodium (Roche, 1 g) 30 minutes before surgery and intravenous infusion of ceftriaxone sodium 1 g twice a day for 3 days after surgery. Deep vein thrombosis was prevented postoperatively, and the patient started subcutaneous injection of enoxaparin sodium (4000 IU produced by Sanofi, once daily for 5 consecutive days) within 24 hours after surgery. The patient was instructed to begin non-weight-bearing functional exercises 48 hours after surgery, including straight leg raising and joint range of motion exercises, with a gradual transition to partial and full weight-bearing exercises.

### Baseline data collection

(1) Demographic characteristics: Age, gender, height, weight, and BMI of the patient. Age, gender were reported by the patients, height and weight were uniformly measured by the research team, height was accurate to 0.1 cm, and weight was accurate to 0.1 kg. BMI was calculated based on the formula weight (kg)/ height (m^2^). (2) Medical history: The clinical history of the patient was collected, focusing on the etiology of ONFH (such as long-term hormone use, alcohol abuse or trauma history), duration of onset, and presence of comorbidities of chronic diseases such as hypertension, diabetes, and coronary heart disease. Medical history was provided by the patient and confirmed by questionnaires and medical records. (3) Imaging data: The degree of ONFH was assessed preoperatively by frontal and lateral X-ray films and MRI of the hip. X-rays were examined by a radiologist and staged according to ARCO staging criteria, and MRI images were evaluated by a specialist. The imaging examination equipment was Philips Achieva 1.5T MRI and digital X-ray machine, and all images were independently viewed by two doctors to ensure data consistency.

### Observation indicators

(1) Oxidative stress-related indicators: Reactiveoxygen species (ROS): ROS are important markers of oxidative stress, reflecting the changes of redox balance in the body. ROS levels in serum were determined using 2',7'-dichlorodicyanofloxacin diacetate (DCFH-DA). Specifically, after serum was separated from 5 mL of collected venous blood, DCFH-DA solution was added and incubated for 30 minutes at 37°C. Fluorescence intensity was measured using a microplate reader (Thermo Scientific Multiskan FC) at the wavelength of 485/535 nm, and the ROS level was calculated based on the fluorescence intensity unit (RFU). The data of all patients included were preoperative, 1 week, 1 month, 3 months and 12 months after surgery. The elevated ROS levels are considered to be a marker of increased oxidative stress. ② Superoxide dismutase (SOD): SOD is an important antioxidant enzyme in the body, which is responsible for scavenging superoxide radicals and reducing the effects of oxidative stress. The level of SOD in serum was determined by enzyme-linked immunosorbent assay (ELISA). The specific operation was to add serum into ELISA plate wells. Absorbance (450 nm wavelength) was measured by ELISA reader using SOD antibody. The frequency of detection was consistent with ROS. ③ Malondialdehyde (MDA): MDA is the end product of lipid peroxidation and a common marker of oxidative stress. Determine the MDA level in serum by colorimetric method by reacting the serum with TBA reagent and measuring the absorbance by spectrophotometer at 532 nm. The frequency of detection was consistent with SOD. 6 months after surgery, ROS level did not increase significantly, SOD level did not decrease significantly, and MDA level did not increase significantly, which was divided into low oxidative stress group. 6 months after surgery, the levels of ROS, SOD and MDA changed significantly compared with those before surgery, suggesting that oxidative stress was enhanced and divided into high oxidative stress group.

(2) Chondrocyte apoptosis rate: All patients' data included articular fluid samples preoperative, 1 week, 1 month, and 3 months after surgery were analyzed by Annexin V-FITC/PI double staining method and flow cytometry (BD Biosciences, Model: FACSCalibur). The proportion of apoptotic cells was detected by flow cytometry to calculate the apoptotic rate of chondrocytes. An increase in the apoptotic rate usually means that the injury of chondrocytes is aggravated. If the apoptotic rate is significantly increased within one month after surgery, it may be related to the postoperative inflammatory response and oxidative stress.

(3) Joint functional recovery: ① VAS score: Hip joint pain was assessed on a 0-10 scale based on the patient's self-reported pain perception (0 indicated no pain and 10 indicated the most severe pain). The data of all patients included were preoperative, 1 month, 3 months and 6 months after surgery. ② Harris hip score: The Harris Score system comprehensively evaluates the hip joint function, motion range, and pain degree. The scoring range was 0-100 points, with 100 points indicating optimal function. The frequency of assessment was consistent with VAS. Harris score above 80 points was considered as excellent, with scores of 70-79 points as good, scores of 60-69 points as medium and scores of below 60 as poor.

(4) Incidence of postoperative complications: The detection of postoperative complications included infection, prosthesis loosening, thrombosis, and nerve injury. All patients' data were examined once a month after surgery, and postoperative complications were recorded and managed in a timely manner. If the incidence of postoperative complications exceeds 10%, it indicates that there is a certain risk in postoperative management.

### Statistical analysis

Statistical analysis was performed using SPSS 25.0 statistical software. Measurement data were expressed as mean±standard deviation (x̄±s), enumeration data were expressed as frequency and percentage, and intra-group comparisons were performed using i-test and chi-square test (*χ^2^*). All data were subjected to normality test, and data conforming to normal distribution were subjected to parametric test. The *Pearson* correlation was used to analyze the correlation between the apoptotic rate of chondrocytes and oxidative stress factors (ROS, SOD and MDA). The correlation coefficient (*R* value) was calculated based on the pre-operative and post-operative mean test data at each time point. The *R* value close to 1 or -1 indicates a strong correlation, and the *R* value close to 0 indicates no correlation. The relevant risk factors for postoperative failure were screened using Log-rank test, including age, gender, medical history, oxidative stress factor level, chondrocyte apoptosis rate and postoperative complications. To further evaluate the independent predictive effect of multiple factors on postoperative failure by Cox regression analysis. Postoperative failure was defined as persistent pain (VAS score >4 for more than 3 months), poor functional recovery (Harris score <70 at 6 months postoperatively), or postoperative complications such as prosthesis loosening or infection. *P*<0.05 indicated that the difference was statistically significant.

## Results

### Baseline data

Analysis of baseline data shows that demographic characteristics such as age, gender, height, weight, and BMI of patients have no significant difference (*P*>0.05) (Table 1). Detailed comparison of key clinical variables related to ONFH severity showed similar distributions between the low and high oxidative stress groups. Specifically, ARCO staging (stage II: 33.8% *vs.* 33.3%; stage III: 41.2% *vs.* 40.9%), lesion size (average necrotic angle: 192.6° ± 45.8° *vs.* 198.3° ± 47.2°), lesion location (medial: 23.5% *vs.* 25.8%; lateral: 30.9% *vs.* 28.8%; central: 45.6% *vs.* 45.4%), degree of femoral head collapse (average: 2.6 ± 1.3 mm *vs.* 2.8 ± 1.5 mm), and pre-operative pain/function scores (VAS: 7.45 ± 1.36 *vs.* 7.78 ± 1.52; Harris: 57.23 ± 13.42 *vs.* 53.87 ± 14.03) were comparable (*P*>0.05). Medical history and imaging data also showed no significant differences, indicating that the basic conditions of the two groups of patients were similar and comparable.

**Table 1:** Baseline data of patients. Note: *P*<0.05 indicated significant difference.

Factor	Result	Standard deviation	Proportion	* t/χ^2^*	* P *
Age (years)	56.92±7.24	7.24	-	0.312	0.755
Gender (Male/Female)	88/46	-	-	0.486	0.485
Height (cm)	167.65±6.32	6.32	-	0.421	0.673
Body weight (kg)	73.04±8.89	8.89	-	0.219	0.827
BMI (kg/m^2^)	26.42±3.23	3.23	-	0.574	0.566
Etiology of ONFH	-	-	-	-	-
Long-term use of hormones	-	-	43 (32.08%)	-	-
Drink excessively	-	-	29 (21.64%)	-	-
Trauma history	-	-	22 (16.42%)	-	-
Other	-	-	40 (29.85%)	-	-
Complication	-	-	-	-	-
Hypertension	-	-	38 (28.36%)	-	-
Diabetes	-	-	23 (17.16%)	-	-
Coronary heart disease	-	-	15 (11.19%)	-	-
Other diseases	-	-	12 (8.96%)	-	-
Duration of onset (Year)	4.56±2.34	2.34	-	-	-
Imaging data	-	-	-	-	-
X-ray film ARCO staging	-	-	-	-	-
ARCO phase I	-	-	10 (7.46%)	-	-
ARCO phase II	-	-	45 (33.58%)	-	-
ARCO phase III	-	-	55 (41.04%)	-	-
ARCO phase IV	-	-	24 (17.91%)	-	-
MRI evaluation	-	-	-	-	-
Mild femoral head injury	-	-	49 (36.57%)	-	-
Moderate femoral head injury	-	-	58 (43.28%)	-	-
Severe femoral head injury	-	-	27 (20.15%)	-	-

### Related indicators of oxidative stress

**Figure 1:**
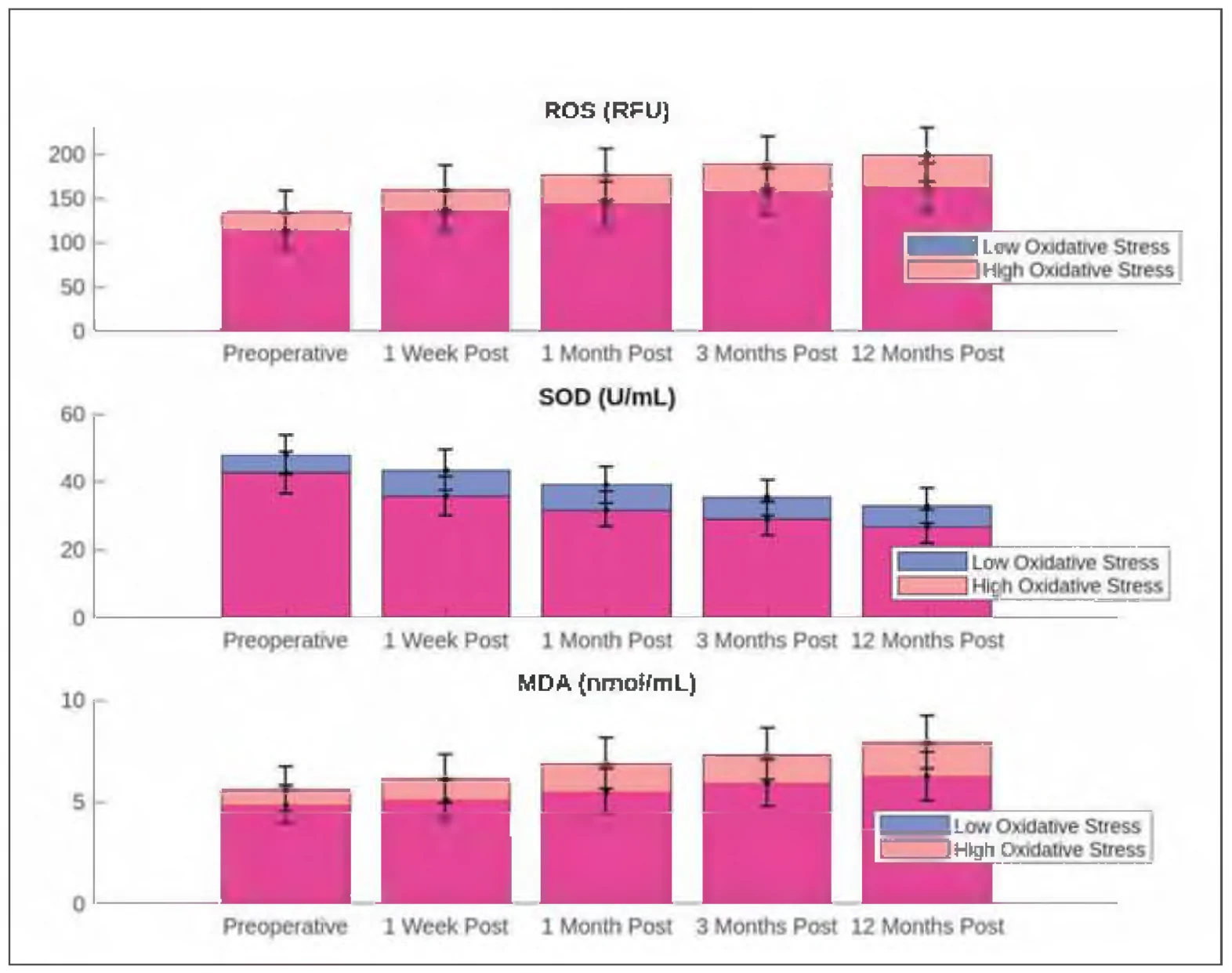
Difference of related indexes of oxidative stress at different time points.

ROS level in the high oxidative stress group was significantly higher than that in the low oxidative stress group (*P*<0.05), while SOD activity was significantly lower than that in the low oxidative stress group (*P*<0.05). Meanwhile, the MDA level in the high oxidative stress group was significantly higher than that in the low oxidative stress group (*P*<0.05) (Table 2, Figure 1).

**Table 2:** Detection results of oxidative stress-related indicators in patients. Note: *P*<0.05 indicated significant difference.

Time point	Group	ROS<br>(RFU)	SOD<br>(U/mL)	MDA<br>(nmol/mL)	* F *	* P *
Preoperative	Low oxidative <br>stress group	112.54±20.34	47.85±5.98	4.85±0.94	-	-
	High oxidative <br>stress group	134.26±23.45	42.53±6.21	5.62±1.10	-	-
1 week <br>after surgery	Low oxidative stress <br>group	135.64±24.01	43.26±6.12	5.12±1.02	4.317	0.032
	High oxidative stress <br>group	159.43±27.32	35.58±5.63	6.14±1.19	5.126	0.018
1 month <br>after surgery	Low oxidative stress <br>group	142.78±25.45	38.95±5.47	5.49±1.11	5.823	0.011
	High oxidative stress <br>group	176.35±29.23	31.75±5.14	6.88±1.24	7.654	0.008
3 months <br>after surgery	Low oxidative stress <br>group	156.83±26.52	35.20±5.31	5.92±1.16	6.032	0.01
	High oxidative stress <br>group	189.21±30.18	29.10±4.85	7.35±1.27	8.342	0.005
12 months <br>after surgery	Low oxidative stress <br>group	162.35±27.45	32.83±5.06	6.25±1.20	7.238	0.007
	High oxidative stress <br>group	198.76±31.25	26.78±4.92	7.92±1.32	9.472	0.002

### Apoptosis rate of chondrocytes

**Figure 2:**
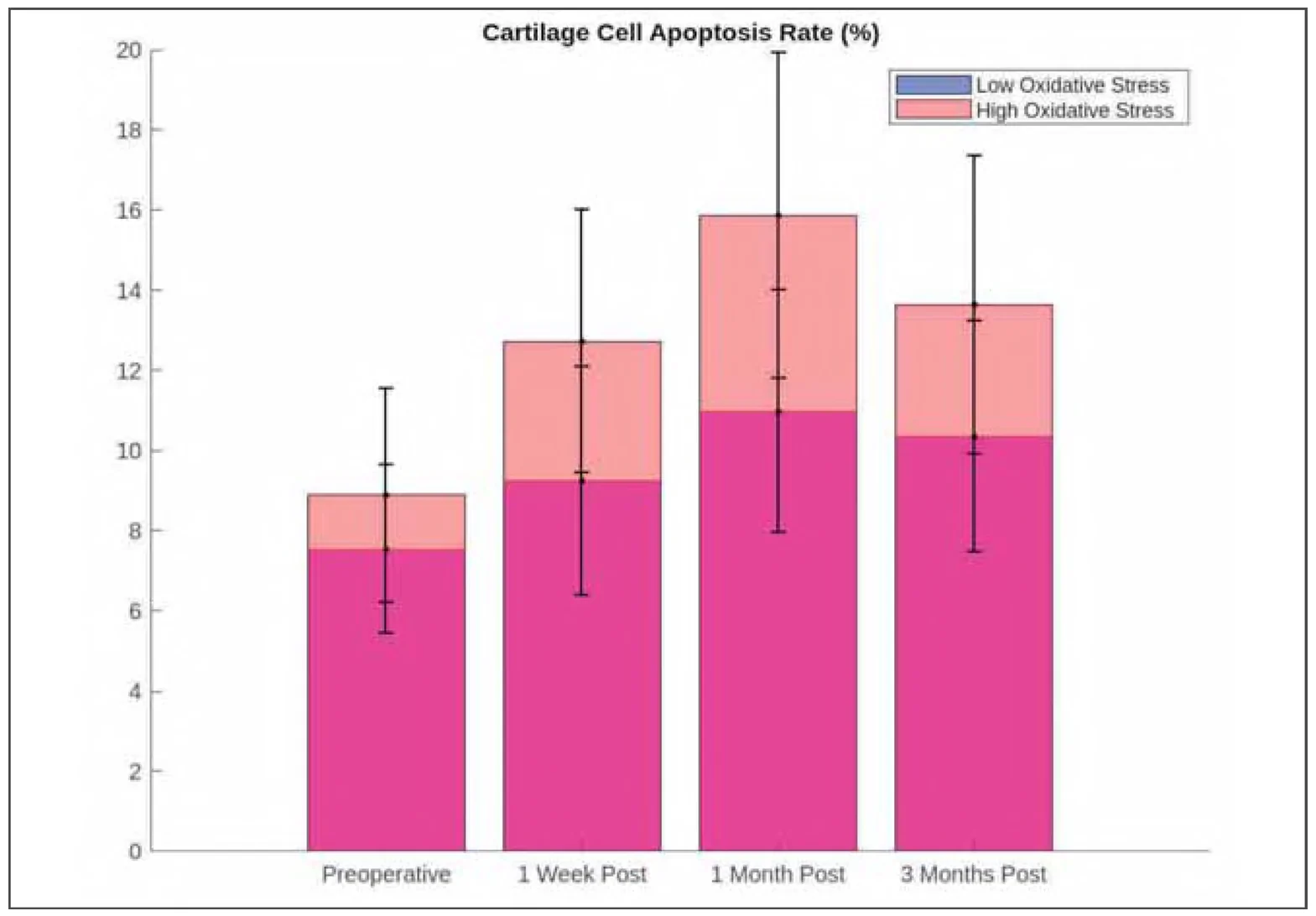
Difference of detection results of apoptotic rate of chondrocytes in different groups.

The apoptosis rate of chondrocytes in the high oxidative stress group was significantly higher than that in the low oxidative stress group at 1 week and 1 month after surgery (*P*<0.05), suggesting that the high level of oxidative stress after surgery might aggravate cell damage (Table 3, Figure 2).

**Table 3:** Detection results of chondrocyte apoptosis rate in patients. Note: P<0.05 indicated significant difference.

Time point	Group	Chondrocyte apoptosis<br>rate (%)	* t *	* P *
Preoperative	Low oxidative stress group	7.53±2.10	-	-
	High oxidative stress group	8.89±2.67	-	-
1 week after surgery	Low oxidative stress group	9.24±2.85	2.115	0.038
	High oxidative stress group	12.73±3.29	4.248	0.015
1 month after surgery	Low oxidative stress group	10.98±3.02	3.327	0.022
	High oxidative stress group	15.88±4.06	6.892	0.004
3 months after surgery	Low oxidative stress group	10.35±2.88	3.174	0.026
	High oxidative stress group	13.65±3.72	5.784	0.008

### Joint function recovery

The VAS score of the low oxidative stress group was significantly lower than that of the high oxidative stress group (*P*<0.05), while the Harris score was significantly higher than that of the high oxidative stress group (*P*<0.05), suggesting that the pain relief after surgery in the low oxidative stress group was more significant, and the joint function recovery was more ideal (Table 4).

**Table 4:** Detection results of joint function recovery in patients. Note: P<0.05 indicated significant difference.

Time point	Group	VAS score	* t *	* P *	Harris score	* t *	* P *
Preoperative	Low oxidative <br>stress group	7.45±1.36	-	-	57.23±13.42	-	-
	High oxidative <br>stress group	7.78±1.52	-	-	53.87±14.03	-	-
1 month <br>after surgery	Low oxidative <br>stress group	5.98±1.85	3.254	0.021	68.23±11.22	4.221	0.015
	High oxidative <br>stress group	6.78±2.04	4.107	0.017	62.41±12.14	5.324	0.009
3 months <br>after surgery	Low oxidative <br>stress group	4.32±1.73	5.132	0.008	74.35±9.98	6.435	0.003
	High oxidative <br>stress group	5.12±1.94	6.487	0.005	68.14±10.45	8.562	0.001
6 months <br>after surgery	Low oxidative <br>stress group	2.56±1.15	8.546	0.002	79.43±8.12	10.219	0
	High oxidative <br>stress group	3.45±1.38	9.762	0.001	72.58±9.34	11.453	0

### Postoperative complications

**Figure 3:**
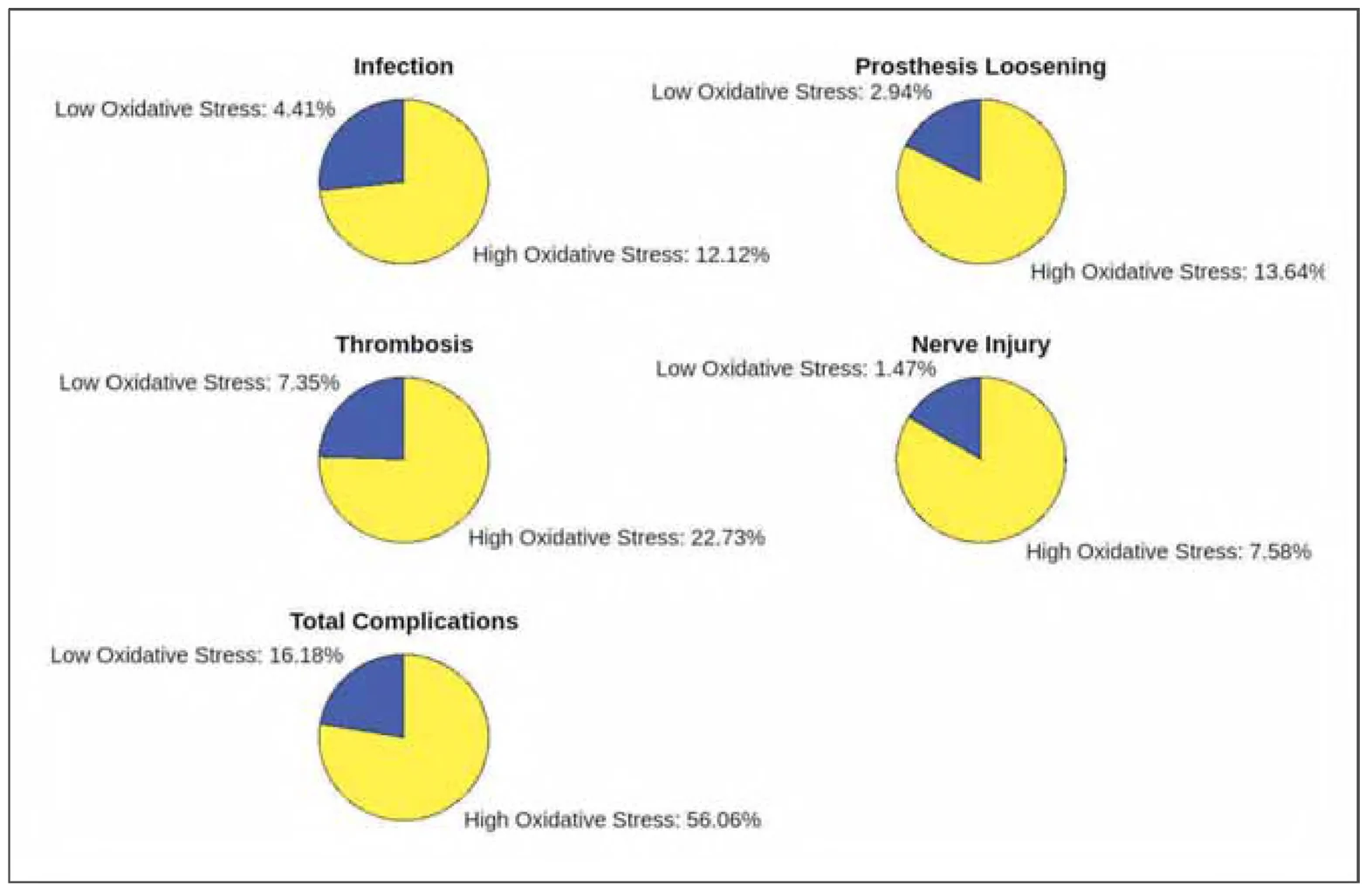
Differences in postoperative complications among different groups.

The incidence of prosthesis loosening, thrombosis and total complications in the high oxidative stress group were significantly higher than those in the low oxidative stress group (*P*<0.05), suggesting that postoperative oxidative stress might be an important risk factor for complications (Table 5, Figure 3).

**Table 5:** Detection results of postoperative complications in patients. Note: P<0.05 indicated significant difference.

Group	Low oxidative stress group<br>(n= 68)	High oxidative stress group<br>(n = 66)	χ^2^	P
Infection n (%)	3 (4.41%)	8 (12.12%)	2.941	0.086
Prosthesis loosening n (%)	2 (2.94%)	9 (13.64%)	4.732	0.030
Thrombosis n (%)	5 (7.35%)	15 (22.73%)	5.896	0.015
Nerve inj'ury n (%)	1 (1.47%)	5 (7.58%)	2.356	0.125
Total complications n (%)	11 (16.18%)	37 (56.06%)	21.457	<0.001

### Pearson correlation analysis

**Figure 4:**
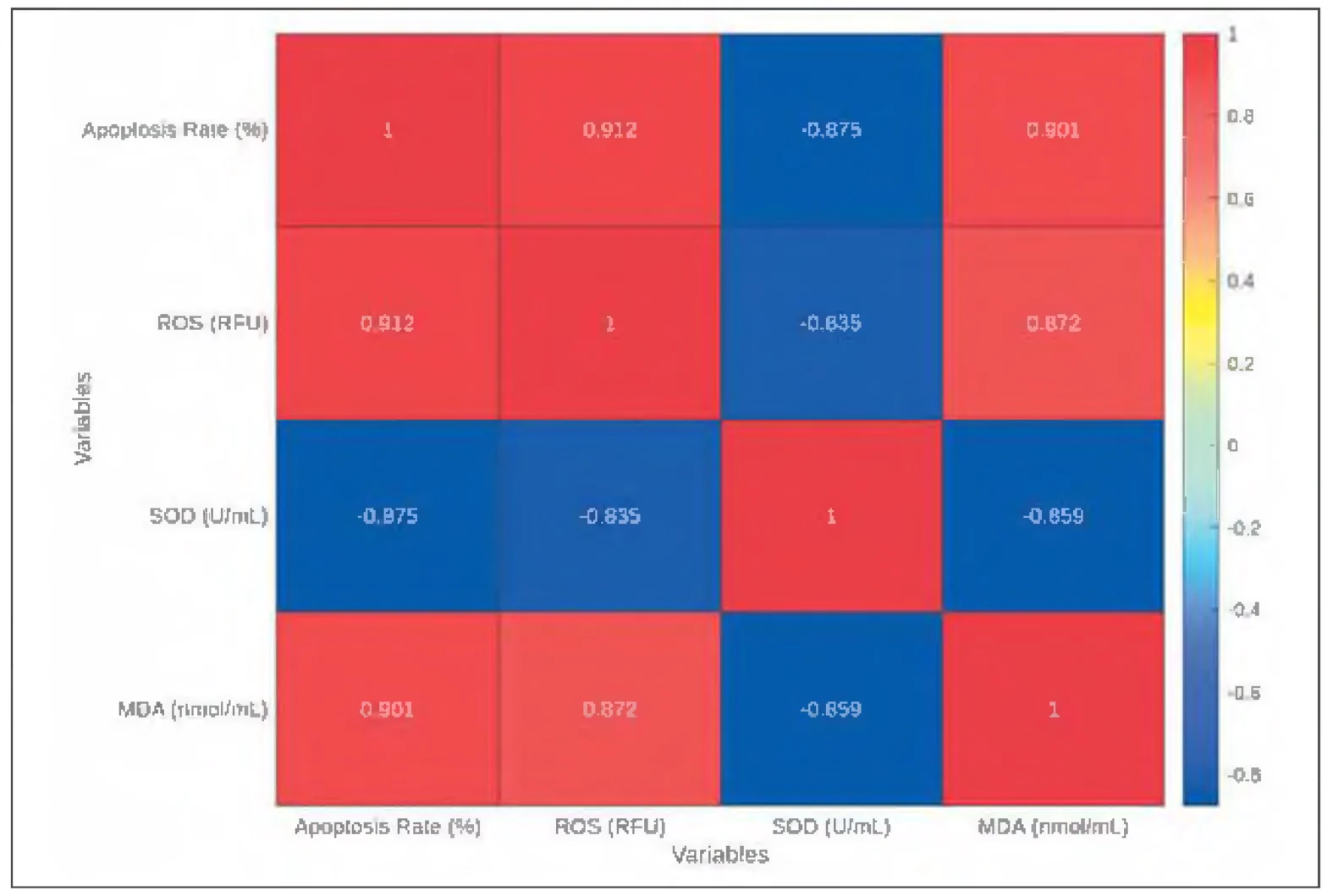
Correlation matrix.

There was a positive correlation between the apoptosis rate of chondrocytes and ROS (*r*=0.912, *P*<0.05), indicating that oxidative stress level had a significant positive correlation with chondrocyte apoptosis rate. The correlation between the chondrocyte apoptosis rate and SOD was negative (*r*=-0.875, *P*<0.05), showing an inverse relationship between the redox state and the chondrocyte apoptosis rate. There was a positive correlation between the apoptotic rate of chondrocytes and MDA (*r*=0.901, *P*<0.05), suggesting that the degree of oxidative stress was closely related to chondrocyte apoptosis (Table 6, Figure 4).

**Table 6:** *Pearson* correlation analysis results. Note: *P*<0.05 indicated significant difference.

Project	Apoptosis Rate (%)	ROS (RFU)	SOD (U/m L)	MDA (nmol/mL)
Apoptosis Rate (%)	1	0.912	-0.875	0.901
Sig. (2-tailed)	-	<0.001	<0.001	<0.001
R O S(RFU )	0.912	1	-0.835	0.872
Sig. (2-tailed)	<0.001	-	0.001	<0.001
SOD (U/m L)	-0.875	-0.835	1	-0.859
Sig. (2-tailed)	<0.001	0.001	-	<0.001
MDA (nmol/mL)	0.901	0.872	-0.859	1
Sig. (2-tailed)	<0.001	<0.001	<0.001	-

### Single factor analysis

Postoperative failure was closely related to age, medical history, oxidative stress factors (ROS, SOD, and MDA), apoptosis rate of chondrocytes, and postoperative complications. In particular, medical history, oxidative stress level, chondrocyte apoptosis rate, and postoperative complications have all shown significant statistical differences, suggesting the important role of these factors in postoperative recovery (Table 7).

**Table 7:** Screening of risk factors related to postoperative failure. Note: *P*<0.05 indicated significant difference.

Risk factor	Failure incidence (%)	Preoperative/Postoperative group<br>comparison (Log-rank P-value)
Age	18 -40 years: 12.3%<br>Over 40 years: 27.5%	0.037
Gender	Male: 22.1%<br>Female: 18.4%	0.465
Medical/case history	With medical history: 30.1%<br>Without medical history: 19.3%	0.012
ROS level	High level: 28.4%<br>Low level: 15.6%	0.023
SOD level	High level: 14.9%<br>Low level: 29.3%	0.041
MDA level	High level: 25.6%<br>Low level: 18.2%	0.031
Chondrocyte apoptosis rate	High apoptosis rate: 30.5%<br>Low apoptosis rate: 16.7%	0.014
Postoperative complications	With complications: 35.0%<br>Without complications: 18.9%	0.009

### Cox regression analysis

Cox regression analysis showed that the independent predictors of postoperative failure included age, medical history, ROS level, SOD level, MDA level, chondrocyte apoptosis rate, and postoperative complications. Postoperative complications were the strongest independent predictors (*HR*=1.489, *P*=0.004), followed by medical history and ROS level (Table 8).

**Table 8:** Independent prediction effect of multi-factors on postoperative failure. Note: *P*<0.05 indicated significant difference.

Risk factor	Regression<br>coefficien(*β*)	Standard error<br>(*SE*)	Risk ratio<br>(*HR*)	95%*CI*	*P*
Age	0.124	0.045	1.132	1.036-1.238	0.009
Gender (Male)	0.158	0.112	1.171	0.939-1.463	0.191
Medical/case history	0.235	0.102	1.266	1.035-1.549	0.029
ROS level	0.055	0.023	1.057	1.010-1.106	0.015
SOD level	-0.092	0.041	0.912	0.844-0.987	0.039
MDA level	0.127	0.054	1.135	1.022-1.260	0.021
Chondrocyte apoptosis rate	0.145	0.061	1.156	1.026-1.303	0.021
Postoperative complications	0.398	0.131	1.489	1.141-1.943	0.004

## Discussion

This study investigated the relationship between oxidative stress markers, chondrocyte apoptosis, and postoperative functional recovery in patients with ONFH undergoing HRA. By integrating clinical outcomes with laboratory indices, we extend previous work and provide a stronger biochemical basis for evaluating recovery and complications in ONFH [11].

Our findings confirmed that serum ROS, SOD, and MDA levels changed significantly within six months after surgery, indicating an altered redox balance. When compared with earlier reports, our study demonstrated that increased oxidative stress is more directly linked to chondrocyte apoptosis and delayed joint function recovery [12]. Elevated ROS and MDA, together with reduced SOD, clearly reflected the aggravation of oxidative damage, thereby influencing cartilage repair and patient prognosis [13]. These results are consistent with prior evidence connecting oxidative stress to articular cartilage injury and postoperative outcomes [14], further supporting oxidative stress as a key biochemical pathway in ONFH pathophysiology [15].

A notable contribution of this study is the mechanistic link between early apoptosis of chondrocytes and later orthopedic complications, such as prosthesis loosening and thrombosis. Early apoptosis, detected at 1 week and 1 month postoperatively, may act as a biological indicator of an unfavorable local microenvironment with excessive oxidative stress. The apoptosis-rich milieu likely promotes inflammatory mediator release, compromises regenerative capacity, and activates coagulation cascades, which together explain why early biochemical alterations precede later clinical complications. This temporal relationship highlights the prognostic value of apoptosis as a surrogate biomarker for long-term surgical outcomes.

The elevated apoptotic rate of chondrocytes observed in our patients aligns with prior reports, reinforcing the role of oxidative stress in aggravating cell damage. Compared with traditional morphological evaluation, our application of flow cytometry provides a more accurate and quantifiable biochemical assessment [16], allowing clinicians to identify risk earlier and tailor interventions to reduce loss of joint function [17].

From the perspective of clinical recovery, pain reduction and improved Harris hip scores confirmed that HRA is effective for restoring function in ONFH [18]. However, the complication rate, especially when exceeding 10%, underscores the need for improved perioperative management strategies [19]. Importantly, Cox regression analysis revealed that although complications were the strongest independent predictor of failure, oxidative stress markers and apoptosis also carried independent prognostic weight. This suggests a sequential biochemical cascade in which oxidative imbalance initiates apoptosis, leading ultimately to complications and failure. Thus, oxidative stress is best understood as an upstream mediator and potential therapeutic target rather than a passive byproduct of surgical outcomes.

The clinical utility of our findings lies in the ability to translate biochemical monitoring into risk stratification. Measuring oxidative stress markers such as ROS, SOD, and MDA, along with apoptosis indices, could allow physicians to identify high-risk patients earlier and initiate targeted interventions. For instance, persistent oxidative imbalance may signal impending cartilage damage or chronic inflammatory activity, warranting antioxidant therapy or intensified rehabilitation. The association between high oxidative stress and specific complications (prosthesis loosening and thrombosis) observed in our study supports the prognostic value of these biomarkers for postoperative risk assessment [20][21][22].

Finally, while correlations are strong, it remains necessary to clarify whether oxidative stress directly drives poor outcomes or reflects underlying comorbidities. Our adjusted Cox regression model accounted for key confounders—including age, comorbid diseases, BMI, preoperative function, and intraoperative factors—yet oxidative stress markers still demonstrated independent predictive value. This strengthens the case for their role as clinically relevant biomarkers.

Overall, the present study emphasizes that biochemical monitoring of oxidative stress and apoptosis can provide clinicians with early and objective indicators of postoperative prognosis. Identifying patients with elevated oxidative stress and apoptosis enables timely interventions, such as antioxidant therapy and structured rehabilitation, thereby improving recovery and quality of life in ONFH [23].

## Conclusion

In conclusion, this study demonstrates that oxidative stress markers (ROS, SOD, and MDA) and chondrocyte apoptosis are strongly associated with postoperative recovery and complication risk in ONFH patients undergoing HRA. Elevated oxidative stress and apoptosis were independent predictors of poor functional outcomes, underscoring their value as prognostic biomarkers. By shifting the focus from purely surgical outcomes to measurable biochemical indicators, our findings provide a laboratory-based approach for early identification of high-risk patients. Monitoring oxidative stress and apoptosis could guide timely interventions, such as antioxidant therapy and tailored rehabilitation, ultimately improving recovery and long-term joint function.

## Dodatak

### Ethical approval

Not applicable.

### Ethical statement

This retrospective study was conducted at Xiamen Chang Gung Hospital in China using deidentified clinical data from existing patient records. All patient identifiers were removed prior to analysis, ensuring confidentiality and compliance with privacy regulations. The study exclusively analyzed anonymized datasets, eliminating risks associated with personal information disclosure. The procedures described, including joint fluid sampling and postoperative monitoring, followed standard clinical protocols with strict adherence to aseptic techniques, minimizing infection risks. The necessity and clinical value of this research—such as identifying early postoperative complications (e.g., infections, inflammatory responses) and improving intervention strategies— significantly outweighed minimal procedural risks. Regular monitoring of joint fluid provided comprehensive biological data critical for understanding postoperative joint microenvironment changes and their correlation with patient outcomes. By enabling early detection of oxidative stress and chondrocyte apoptosis, this study supports timely clinical interventions to reduce complication rates. As the data were retrospective and fully anonymized, institutional review board (IRB) approval was not mandated under U.S. federal regulations (45 CFR 46) for secondary use of de-identified health information. This approach aligns with ethical guidelines prioritizing patient privacy while advancing scientific knowledge to enhance postoperative care for osteonecrosis patients.

### Acknowledgements

As no corresponding research cases were found in the author's country, the case data included in this study were provided by several major medical institutions in the eastern United States. The authors sincerely acknowledge Xiamen Chang Gung Hospital for providing the clinical datasets and supporting facilities for this retrospective study. Although these hospitals are not listed in the authorship due to institutional confidentiality and data-sharing protocols, their collaboration was essential to the completion of this research. The comprehensive patient records, including preoperative and postoperative oxidative stress indicators, chondrocyte apoptosis rates, and functional recovery metrics, enabled a robust analysis of risk factors and outcomes following hip resurfacing arthroplasty.

We extend our gratitude to the clinical teams and data custodians at these institutions for their meticulous efforts in de-identifying patient information and ensuring compliance with privacy regulations under U.S. federal guidelines (45 CFR 46). Their commitment to ethical data practices allowed this study to proceed without compromising patient confidentiality. The anonymized datasets provided critical insights into postoperative complications, such as joint infections and inflammatory responses, which are pivotal for refining clinical intervention strategies.

Additionally, we appreciate the technical support provided by these hospitals in standardizing data collection protocols, including imaging assessments and biomarker analyses. Their rigorous methodology ensured the reliability and comparability of results across diverse patient cohorts. This collaborative effort underscores the importance of international data-sharing initiatives in advancing orthopedic research, particularly for conditions like osteonecrosis of the femoral head, where early detection of oxidative stress and chondrocyte apoptosis can significantly improve surgical outcomes. Finally, we thank the patients whose anonymized data contributed to this study, as their clinical journeys provided the foundation for understanding postoperative recovery dynamics and optimizing future therapeutic approaches.

### Conflict of interest statement

All the authors declare that they have no conflict of interest in this work.

## References

[1] Zhang J, Wan S, Luo X, Zhang C, Wu C, He L, et al (2024). Increasing the angle between caudal screw and the transverse plane may aggravate the risk of femoral head necrosis by deteriorating the fixation stability in patients with femoral neck fracture. Eur J Med Res.

[2] Yong M, Xu M, Lou Y, Lin G (2023). Risk factors for postoperative avascular necrosis of the femoral head in children with developmental dysplasia of the hip. Front Pediatr.

[3] Fang G Z, Lin J, Cao L H, Liu T S, Ma Y H, Yang L (2024). Changes in postoperative depression and anxiety and their relationship with recovery from femoral head necrosis: A longitudinal study. World J Psychiatr.

[4] Manzotti A, Larghi M, Placenza E, Susini F, Grassi M (2021). Postoperative outcomes in total hip arthroplasty following femoral head avascular necrosis in HIV-positive patients. Acta Biomed.

[5] Su J, Zhang Y, Cao B, Li X (2022). Analysis of Factors Influencing Postoperative Femoral Head Collapse in Patients With Ficat I, II, and III Stages of Aseptic Necrosis of the Femoral Head. J Am Acad Orthop Sur.

[6] Yuan Y, Zou M, Wu S, Liu C, Hao L (2024). Recent advances in nanomaterials for the treatment of femoral head necrosis. Hum Cell.

[7] Londhe S B, Khot R, Shah R V, Desouza C (2022). An early experience of the use of dual mobility cup uncemented total hip arhroplasty in young patients with avascular necrosis of the femoral head. J Clin Orthop Trauma.

[8] Li Y, Sun D, Wang K, Liu J, Wang Z, Liu Y (2022). Postoperative avascular necrosis of the femoral head in pediatric femoral neck fractures. PLoS One.

[9] Park B K, Park H, Park K B, Rhee I, Kim S, Kim H W (2022). Fate of hips complicated by avascular necrosis of the femoral head following reconstructive surgery in nonambulatory patients with cerebral palsy. Sci Rep-Uk.

[10] Shoji T, Shozen H, Ueki S, Kaneta H, Yaunaga Y, Adachi N (2024). Evaluation of the long-term patient-reported outcomes after hip arthroplasty or joint preserving with Sugioka femoral osteotomy in patients with femoral head osteonecrosis. Int Orthop.

[11] Li S, Wang J, Ma R, Zhao C, Gao Z, Quan X, et al (2023). Analysis of the efficacy of drilling decompression autologous bone marrow and allogeneic bone grafting in the treatment of HIV-positive patients with early osteonecrosis of the femoral head. Bmc Musculoskel Dis.

[12] Hamada R, Kawano T, Murao M, Nankaku M, Okuzu Y, Kawai T, et al (2024). What are the differences in the recovery of physical function and clinical score between patients with steroid-related osteonecrosis of the femoral head and hip osteoarthritis undergoing total hip arthroplasty? A propensity score-matched study. Int Orthop.

[13] Zhang Z, Chi J, Driskill E, Mont M A, Jones L C, Cui Q (2024). Effect of Patient Age on Total Hip Arthroplasty Outcomes in Patients Who Have Osteonecrosis of the Femoral Head Compared to Patients Who Have Hip Osteoarthritis. J Arthroplasty.

[14] Tran T N, Wolf M, Winter P, Landgraeber S (2022). Hip joint mechanics in patients with osteonecrosis of the femoral head following treatment by advanced core decompression. Clin Biomech (Bristol).

[15] Mei J, Wang S, Ni M, Zhang F, Tang J, Bi G, et al (2022). Association between Weitbrecht's Retinaculum Injury and Femoral Head Necrosis in Femoral Neck Fractures. Orthop Surg.

[16] Necas L, Hrubina M, Melisik M, Cabala J, Cibula Z, Daniel M (2023). Total hip arthroplasty with ultra-short uncemented stem in patients with osteonecrosis of the femoral head: mid-term results. Hip Int.

[17] Wang T, Gao C, Wu D, Li C, Cheng X, Yang Z, et al (2023). One-year unplanned readmission after total hip arthroplasty in patients with osteonecrosis of the femoral head: rate, causes, and risk factors. Bmc Musculoskel Dis.

[18] Chuang F K, Yeh T T, Hung C C, Hsu C L, Shih J T, Shen P H (2023). Effects of malnutrition on outcomes of patients with femoral head osteonecrosis undergoing total hip arthroplasty: A population-based study. Nutr Clin Pract.

[19] Kim H S, Park J W, Ha J H, Lee Y K, Ha Y C, Koo K H (2022). Third-Generation Ceramic-on-Ceramic Total Hip Arthroplasty in Patients with Osteonecrosis of the Femoral Head: A 10- to 16-year Follow-up Study. J Bone Joint Surg Am.

[20] Hoggard T M, Chen D Q, Quinlan N D, Bell J E, Werner B C, Cui Q (2022). Outcomes Following Total Hip Arthroplasty for Osteonecrosis of the Femoral Head in Patients on Hemodialysis. J Bone Joint Surg Am.

[21] Lyu J, Ma T, Huang X, Shi J, Huang G, Chen F, et al (2023). Core decompression with beta-tri-calcium phosphate grafts in combination with platelet-rich plasma for the treatment of avascular necrosis of femoral head. Bmc Musculoskel Dis.

[22] Song Y S, Lee W W, Park M S, Kim N T, Sung K H (2022). Usefulness of Bone SPECT/CT for Predicting Avascular Necrosis of the Femoral Head in Children with Slipped Capital Femoral Epiphysis or Femoral Neck Fracture. Korean J Radiol.

[23] Palekar G, Bhalodiya H P, Archik S, Trivedi K (2021). Retrospective Study on Implantation of Autologous-Cultured Osteoblasts for the Treatment of Patients with Avascular Necrosis of the Femoral Head. Orthop Res Rev.

